# Overconfidence and Career Choice

**DOI:** 10.1371/journal.pone.0145126

**Published:** 2016-01-25

**Authors:** Jonathan F. Schulz, Christian Thöni

**Affiliations:** 1 Department of Psychology, Yale University, New Haven, Connecticut, United States of America; 2 University of Lausanne, Lausanne, Switzerland; Middlesex University London, UNITED KINGDOM

## Abstract

People self-assess their relative ability when making career choices. Thus, confidence in their own abilities is likely an important factor for selection into various career paths. In a sample of 711 first-year students we examine whether there are systematic differences in confidence levels across fields of study. We find that our experimental confidence measures significantly vary between fields of study: While students in business related academic disciplines (Political Science, Law, Economics, and Business Administration) exhibit the highest confidence levels, students of Humanities range at the other end of the scale. This may have important implications for subsequent earnings and professions students select themselves in.

## Introduction

Laboratory experiments show that confidence is an important factor for selection (e.g. [1–3]). However, it is less well understood how these results extrapolate to a real world setting such as career choice. Choosing the field of study is certainly an important–if not the most important–career decision in life. It has a strong influence on a person’s professional choices later in life, and earnings depend to a great extent on students’ major [4]. To the degree that confidence drives selection into academic disciplines, it affects career paths and earnings. Confidence may also explain gender differences in labor-market outcomes. Several studies find that females are less confident than males (see e.g. [5] or [6]).

In our study we relate first year students’ field of study to an experimental confidence measure. We find considerable differences: On average, students from Political Science, Law, Economics and Business Administration are overconfident, while students from Humanities tend to be underconfident. This finding is consistent with a selection interpretation. While we cannot rule out that this heterogeneity may also be driven by exposure to certain fields of study, regression analysis does not support this interpretation. The duration students are exposed to a field of study does not seem to have a systematic impact on confidence levels.

Independent of the origins of the observed heterogeneity, consequences may not only follow for individuals, but also for whole professions. Graduates from certain academic disciplines might be on average more prone to the many phenomena overconfidence is associated with like excess entry into markets [1,2], value-destroying mergers [7,8], excessive job market search and unemployment [9], or frictions and inefficiencies in financial markets [10–13]. Our data suggests that students, who major in disciplines generally taught at business schools, are more likely to be overconfident. As many of them will later work in influential management positions, employers may want to take overconfidence into account.

Related to our study is Niederle and Vesterlund [3]. In laboratory settings they find that selection into competitive environments is partly explained by differences in confidence levels (as well as competitive preferences): men exhibit greater preference for competition as well as greater confidence in their ability than women and are more likely to select themselves into competitive environments. These differences can already be found for 9 to 18 year olds [14]. While these studies find that women have an inherently lower willingness to compete, [15] and [16] find that gender effects are mainly driven by differences in confidence. Because we do not elicit a measure for cooperative preferences, our data does not allow to investigate the separate influence of competitive preferences and confidence on the outcome. Our results suggest that overconfidence is a predictor for a students’ field of study, an effect which might be mediated by preferences for competition.

Closest to our work is Buser et al. [17], who focus on the extrapolation of [3]. They show that competitive preferences are significantly linked to curriculum choices of 15 year old high school students in the Netherlands: Students with stronger preferences for competition are more likely to select into more prestigious curricula. In contrast to our study their main focus is on competitive preferences. They do not find evidence for a separate effect of confidence on curriculum choice. Our study focuses at individuals at a later state of their educational career. Compared to the four curricula high school students choose from, the choice of a field of study is presumably more decisive with regard to future occupations and career paths.

## Experimental Design and Procedures

We conducted our study with first year students and focused on relative ability judgments. In a laboratory environment we measure individual confidence in a very intuitive and incentive compatible way. Subjects are ranked according to their performance in trivia questions and subsequently guess their rank within a well-defined group of participants. Trivia questions as performance measure have been widely used [1,18–20].

**Performance measure:** participants guess the year of five historical events of the 20^th^ century (S1 File). We choose the events such, that students should know them, but uncertainty to the exact year remains. For correctly answering a question subjects earn 2 Swiss Francs; for each year the answer deviates 0.2 Francs are deducted (deviations of 10 or more years yield no payoff). We define the performance of a subject as the sum of absolute deviations from the correct answers across all questions.

**Ranking:** subjects are asked to rank themselves within their benchmark group. The benchmark group consists generally of twelve individuals who were all present in a particular session. Since we were interested whether our confidence measure is robust to the group size subjects rank themselves in, we ran some sessions where subjects ranked themselves in groups of 6, to 36 participants (group sizes were varied in session at both locations the experiment was conducted). For the analysis we rescale all ranks to the interval between 1 and 12. We do not find any significant difference regarding group size. All group sizes result in similar averages and standard deviations for the normalized measures, and in the estimates reported below we control for group size and find no indication for systematic effects. For estimating their correct rank subjects receive five Francs, any other estimate is not rewarded. To rule out hedging subjects are informed about this rank guessing task only after they completed the trivia quiz.

**Confidence measure:** The difference between a subject’s rank estimate and its true rank constitutes our measure of confidence. In the data analysis we make use of all information and calculate true ranks based on the entire sample of a given cohort. An accurate estimate of the own relative performance corresponds to zero while positive differences indicate overconfidence.

In total 711 students participated in the experiment, 343 students in 10 sessions at the University of Zurich and 368 students in 18 sessions at the University of St.Gallen. The University of St.Gallen offers five fields of study: Political Science, Law, Economics, Other Social Sciences and Business Administration, while the Zurich sample contains a considerable larger variety of academic disciplines including all fields taught in St.Gallen. Including two subject pools–even though one has a smaller subset of disciplines–gives us an indication on the robustness of the results. It allows us to test whether the same disciplines in different universities have similar levels of confidence. Students were recruited online from a participant pool at each university. At the time we ran the experimental sessions none of the involved universities offered the possibility to obtain an approval from an IRB board. Upon enrolling to the database of experiment participants, students signed a consent form. The consent form informed subjects that the data produced in the experiment would be anonymized and used exclusively for scientific purposes. The experiment lasted about 12 minutes and was added to unrelated experiments lasting in total 1.5 h. Students earnings in this experiment averaged 4.13 Francs (about $4.6). The experiment was computerized and programmed with z-Tree [21].

## Results

Our data reproduces the better-than-average effect from psychological research (even though not nearly as pronounced as in [22]): the majority of subjects (55 percent) judge themselves to be better than the median of 6.5. On average subjects overplace themselves by 0.44 ranks. A Wilcoxon signed-rank test reveals that confidence is significantly different from zero (*z* = 3.412, *p* = 0.0006).

**Fig 1:**
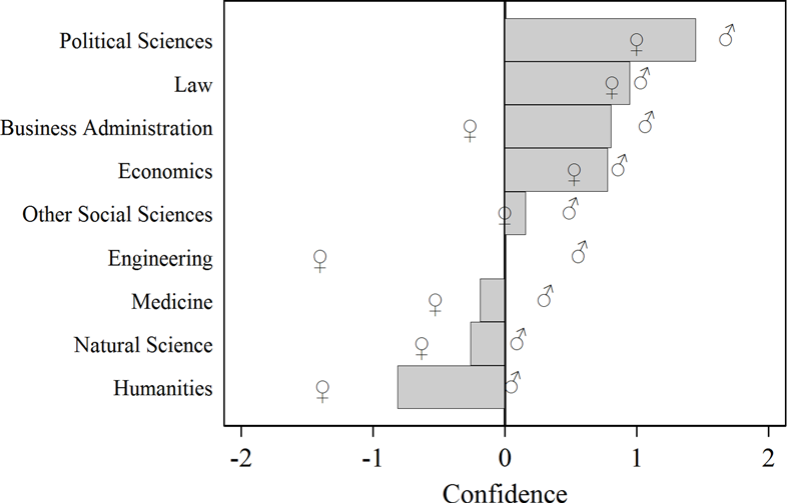
Mean confidence levels by field of study and gender. Bars show mean confidence for both genders. The symbols ♂ and ♀ indicate mean confidence of male and female subjects, respectively. Political Science, Law, Business Administration and Economic students exhibit the highest confidence levels, whereas Humanities, Natural Science, Medicine and Engineering fall at the other end of the scale. To a large extent ordering of disciplines remains the same when looking at each gender separately. Females generally exhibit lower confidence levels.

Fig 1 sets the stage for our main finding. The mean confidence levels by academic discipline reveal a considerable degree of heterogeneity: Political Science, Law, Business Administration and Economic exhibit the highest confidence levels. Students overplace themselves between 1.4 and 0.8 ranks. On the other hand, students of Humanities, Natural Science, or Medicine underestimate their true rank by 0.8, 0.3 and 0.2 ranks respectively. A Kruskal-Wallis test rejects the hypothesis that the confidence levels in different fields of study stem from the same distribution (*χ*^2^(8) = 19.5, *p* = 0.013). These differences exists even though there are no systematic differences in the ability to solve the trivia questions between the fields of studies. A Kruskal-Wallis test does not find significant differences of the actual ranks between the different fields of studies (*χ*^2^(8) = 6.8, *p* = 0.558).

In almost all fields of study we find that females exhibit lower confidence than males. However, it is also apparent that the heterogeneity in academic disciplines is not primarily driven by differences in the gender composition. For each gender there is considerable heterogeneity across academic disciplines. This heterogeneity follows a similar pattern for both genders, with Political Science exhibiting the highest and Humanities the lowest confidence.

Fig 1 also reveals that disciplines that are generally taught in business schools (Political Sciences, Law, Economics and Business Administration) rank higher in confidence than the other fields (Wilcoxon rank sum test, *z* = –4.084, *p* < 0.0001). This is also the case when we test only within the Zurich sample (*z* = –1.99, *p* < 0.05). While we are not able to conduct this test for the University of St.Gallen only (due to the lack of non-business related discipline), we are able to compare the business school related disciplines between Zurich and St.Gallen. This gives insights about the robustness of our results. We find very similar levels of confidence between the two locations among the students from business related disciplines (Zurich: 0.78; St.Gallen: 0.91; *z* = 0.172, *p* = 0.864).

Table 1 reports the coefficients for our confidence measure from multinomial logistic regressions where the dependent variable is the field of study, with Political Science as reference category. In Model (1) we explain selection into discipline with confidence, controlling for gender, the question set, and the true performance. In Model (2) we add a control for the subject pool (dummy for St.Gallen), cohort effects and the size of the reference group. Finally, in Model (3) we add individual controls for age, family background (relative income and number of siblings), and number of subjects known within the session. Wald-tests reject the hypothesis that all coefficients for confidence are simultaneously zero (Model (1): *p* = 0.008, (2): *p* = 0.059, (3): *p* = 0.041). Thus, for example, a person with lower confidence is significantly more likely to study Humanities than Political science. Similarly, testing for differences in the confidence coefficients (in model 1) he is more likely to study Humanities than Law (*p* = 0.049), or Business Administration (*p* = 0.062).

**Table 1:** Field of study and confidence.

Confidence coefficient for	(1)	(2)	(3)
Law	-0.129 (0.097)	-0.131 (0.111)	-0.147 (0.111)
Economics	-0.224*** (0.086)	-0.242** (0.096)	-0.242** (0.096)
Business Administration	-0.188*** (0.066)	-0.209*** (0.074)	-0.212*** (0.075)
Other Social Sciences	-0.250*** (0.097)	-0.265** (0.114)	-0.290** (0.117)
Engineering	-0.267*** (0.092)	-0.282** (0.118)	-0.316*** (0.120)
Medicine	-0.378*** (0.106)	-0.408*** (0.132)	-0.441*** (0.136)
Natural Science	-0.229*** (0.076)	-0.234** (0.106)	-0.267** (0.108)
Humanities	-0.340*** (0.095)	-0.364*** (0.120)	-0.415*** (0.124)
Controls for			
gender, relative performance, question set	Yes	Yes	Yes
subject pool, cohort, group size	No	Yes	Yes
age, relative income	No	No	Yes
N	711	711	711
Pseudo R^2^	0.091	0.278	0.297

*Notes*: Multinomial logistic regressions with robust standard errors. Depended variable is the field of study. We show coefficients (standard errors) for Confidence for each academic discipline. The reference discipline is Political Sciences.

* *p* < 0.1

** *p* < 0.05

*** *p* < 0.01.

Table 1 reveals that confidence is predictive of the field of study. However, the multinomial logit regression in Table 1 does not allow to disentangle whether these findings are due to selection or rather due to the experience in a specific discipline. To test for the latter possibility we report OLS regressions in Table 2, where the dependent variable is confidence, and where we include the variable ‘exposure’ as explanatory variable (in column 1). This variable captures the duration a student was exposed to a field of study (measured as the number of weeks from the start of the semester until the experiment). Further controls are dummy variables for gender, for the subject pool, the field of study, the size of the reference group, the year the study was conducted, the question set and subjects’ actual rank. This last control variable is necessary since the confidence measure contains floor and ceiling effects: e.g. a person having the highest actual rank cannot overestimate his rank. To get a more detailed picture–namely whether there are heterogeneous effects of exposure—we interacted ‘exposure’ with the field of study in column (2).

**Table 2:** Confidence and exposure to field of study.

	(1)		(2)	
	Confidence		Confidence	
	b	se	b	se
Political Sciences	1.607***	(0.537)	2.206*	(1.220)
Law	1.124**	(0.504)	1.190	(1.243)
Economics	0.699	(0.488)	1.304	(1.132)
Business Administration	0.852*	(0.454)	1.392	(1.078)
Other Social Sciences	0.532	(0.459)	0.463	(1.223)
Engineering	0.322	(0.440)	1.669	(1.409)
Medicine	-0.150	(0.495)	0.102	(1.532)
Natural Science	0.590	(0.380)	-0.221	(1.148)
Female	-1.505***	(0.178)	-1.489***	(0.179)
Exposure	0.009	(0.013)		
Exposure x Political Sciences			-0.007	(0.033)
Exposure x Law			0.026	(0.039)
Exposure x Economics			-0.010	(0.028)
Exposure x Business Administration			-0.005	(0.017)
Exposure x Other Social Sciences			0.048	(0.053)
Exposure x Engineering			-0.064	(0.071)
Exposure x Medicine			0.018	(0.089)
Exposure x Natural Science			0.097**	(0.039)
Exposure x Humanities			0.037	(0.074)
Subject pool (U St.Gallen)	-0.271	(0.370)	-0.209	(0.373)
Controls (cohort, group size, actual rank, question set, const.)	Yes		Yes	
Observations	711		711	
*R*^2^	0.646		0.650	

*Notes*: OLS Regression of gender, field of study, subject pool and the exposure to a particular discipline on overconfidence. Standard errors are in parenthesis. We also included controls for the year the study was conducted, the size of the reference group, the question set and subject’s actual rank. Column (3) and (4) only contains data from sessions conducted in Zurich.

* *p* < 0.1

** *p* < 0.05

*** *p* < 0.01

Columns 1 and 2 suggest that the duration a student is exposed to an academic discipline does not systematically influence confidence levels, as the coefficient for the variable ‘exposure’ in column 1 is not significant. In addition, interacting ‘exposure’ with the field of study we do not find evidence that being exposed to Political Science, Law, Business Administration or Economics increases confidence. The only field of study where we find an effect is Natural Science, where the results suggest an increase in confidence.

Table 2 also reveals a pronounced gender effect: females are highly significantly less confident than males. On average their difference between guess and true rank is about a 1.5 units lower. Further, Table 2 (column 1) corroborates the finding of heterogeneity between the fields of studies. Political Science, Law, and Business Administration exhibit higher confidence level than Humanities (our reference category). With the exception of Law, Political Science students exhibit higher confidence than all the other disciplines. Differences in confidence also exist between Medicine on the one hand and Law (*p* = 0.027) and Business Administration (*p* = 0.053) on the other hand. Furthermore, both models reveal that there are no significant differences between the subject pool of the University of Zurich and the University of St.Gallen when controlling for the field of study.

## Discussion and Conclusions

Laboratory experiments have shown that confidence is an important factor for selection into competitive environments (see e.g. [1–3]). Our results corroborate this finding in making a connection to one of the major decisions in an individual’s life: selecting a field of study. We find that a subject’s confidence level is a significant predictor for the choice of academic discipline. While our results are supportive of a selection interpretation, we cannot rule out that subjects’ varying confidence levels are shaped by the experience they gained in their studies. However, our data stems from first year students, who only had limited exposure to an academic discipline. Indeed, we do not find evidence that the duration in a particular field of study drives our results. Evidence that confidence matters when selecting into higher education also comes from Chevalier et al., [23], who find that high school students with a more positive view of their academic abilities are more likely to expect to continue on to higher education even after controlling for observable measures of ability and characteristics.

In our experiment we elicited overconfidence via trivia quizzes. Even though this is a widely used performance measure in the literature on overconfidence (see e.g. [1,19,20]) some caution applies to its generalizability. We have shown that there are no systematic differences in the ability to solve trivia quizzes between the fields of study in our sample. It might still be the case that students from academic disciplines typically taught in business schools (particularly political science students) exhibit high confidence levels in the domain of world affairs in the 20th century because they believe it is in their domain of proficiency. This would accentuate problems associated with overconfidence: it would imply that subjects are particularly overconfident in the domain they self-selected in—even after controlling for actual skill.

Selection into different careers based on confidence has an important impact on an individual’s lifetime earnings, as there are sizeable differences among graduates from various disciplines in starting salaries [4]. Reuben et al. [24] show that overconfident individuals have higher earnings expectations. Indeed, the Swiss graduation survey [25] shows that the high confidence disciplines in our sample are also generally the fields with higher earnings five years after obtaining a master’s degree. For example, averaging median income over the disciplines generally taught at business schools leads to an income of 106’800 Francs compared to 94’100 Francs when averaging over the remaining disciplines. Of course, overconfidence in itself may be favorable to higher incomes. Overconfident individuals may more credibly convince others of their high ability [26–28].

Independent of their origins, be it selection or education in a particular discipline, heterogeneous confidence levels may subsequently have important consequences. Graduates from disciplines that are generally taught in business schools may be relatively more prone to excess entry into markets, value destroying mergers or other phenomena the literature associates with overconfidence. At the same time those graduates may be more likely to overcome self-control problems and therefore commit to more ambitious projects [29].

## Supporting Information

S1 File Experimental material: English translation of the experimental instructions and question sets. (DOCX)
