# Supplementary material for: Overconfidence and Career Choice
Source: PLoS One. 2016 Jan 25;11(1):e0145126. doi: 10.1371/journal.pone.0145126 (PMC4726650; doi:10.1371/journal.pone.0145126)
Supplement: S1 File — (DOCX) [file pone.0145126.s001.docx]

## S1 File: Experimental Material

## Experimental Instructions

## *This is the text that was displayed to the students on the computer screen:*

In this study we ask you for the years of various events in the 20^th^ century. A question could look like this:

In which year did the reunification of Germany take place?

When you fill in the correct answer (1990), you get 2 Franks. If you fill in a wrong answer, your income depends on the absolute deviation from the correct answer in the following way:

First the absolute difference between your number and the correct date is calculated. For every year your answer deviates from the correct solution (not matter if either upward or downward) you get 0.2 Franks less. If you are 10 or more years from the correct answer, you do not receive any remuneration.

For example, if you put in the year 1992 instead of the correct answer, you will get 1.6 Franks since your answers deviates by two years from the correct one.

If you have any questions, please do not hesitate to direct them to us.

*After all subjects participated in the year guessing task instructions for the ranking task were displayed:*

In this part of the study you are randomly allocated to a group of 12 persons in total. All persons in your group have answered the same questions as you.

The computer has created a ranking list of all participants in your group. The ranking is based on the sum of all absolute deviations from the correct year. This measure reveals how well the years were estimated altogether.

In doing so the person with the smallest sum of absolute deviations gets the rank 1, the person with the second smallest deviation the rank 2 and so on. The person with the greatest sum of deviation gets the rank 12.

Before you are informed of your rank you will have to fulfill another task. You have to estimate you rank. As before, you get an amount of money if your estimate is correct. If you estimate your rank exactly, you get an additional 5 Franks.

On the next screen you are requested to state the estimate of you rank. If something is not clear to you, please do not hesitate to ask.

*After that input screen ‘relative rank estimation’ was displayed:*

What rank do you think you occupy (in your group of 12 participants)?

Rank 1: Smallest sum of deviations between estimated and correct years, that is, years were estimated the best.

Rank 2: Greatest sum of deviations between estimated and correct years, that is, years were estimated the least well.

## Question Sets

## Set 1 (n=593)

In which year was the Nobel Prize in physics awarded to Albert Einstein?

In which year was pope Johannes Paul I (the direct predecessor of Johannes Paul II) elected Pope?

In which year did the reactor accident happen in Chernobyl?

In which year was Elvis Presley born?

In which year did the first flight with the supersonic jet Concorde take place?

**Set 2 (n=31)**

In which year was the Israeli State founded?

In which year was John Lennon murdered?

In which year was John F. Kennedy born?

In which year did the first manned space travel take place?

In which year did the Cuban Missile Crises take place?

**Set 3 (n=29)**

In which year was the Israeli State founded?

In which year was Boris Yeltsin elected president of the Soviet constituent republic of Russia?

In which year was John F. Kennedy born?

In which year did the Vietnam War end?

In which year did the Cuban Missile Crises take place?

**Set 4 (n=16)**

In which year did Brazil win the soccer world championship the second time?

In which year did Niki Lauda become the world champion in Formula One for the first time?

In which year did the first Olympic winter games take place?

In which year was the UEFA Champions League introduced?

In which year was Muhammad Ali born?

**Set 5 (n=19)**

The American scientist Robert Noyce patents the computer chip.

Women get the right to vote in the US.

Alexander Fleming discovers the first antibiotic, Penicillin.

Guglielmo Marconi sends the first radio signal across the Atlantic.

Mahatma Gandhi takes over the leadership of the non-violent reform movement in India.

**Set 6 (n=23)**

In what year did Japan attack Pearl Harbor?

In what year was the first cloned sheep born?

In what year did Adolf Hitler become Reich Chancellor?

In what year was the first web-site published?

In what year did Lady Di (Diana Spencer) die?
